# Clinical Society of Maryland

**Published:** 1894-04

**Authors:** H. O. Reik

**Affiliations:** Secretary; 525 N. Howard St.


### CLINICAL SOCIETY OF MARYLAND.

### STATED MEETING HELD FEBRUARY 16TH, 1894.

The twenty-third regular meeting was called to order by the Vice-President, DR. HERBERT HARLAN.

DR. ROBERT W. JOHNSON read a paper on "Strangulation of the Testicle Due to Twist of Spermatic Cord," and exhibited specimen.

DR. W. S. GARDNER followed with a paper on "The Conservative Surgery of the Uterine Appendages."

Last year, in a paper read before the Medical Journal Club, I reviewed the literature bearing upon the treatment of diseases of the appendages, and endeavored to show that a large number of cases of what are now known to be salpingitis were not only relieved but permanently cured by purely medical treatment. The basis of the treatment was that inflammations of the Fallopian tubes, like other inflammations, have a tendency to recovery if the diseased organ is put at rest.

After the advocates of this rational form of treatment came those who have gone so far as to say that all diseased tubes and accompanying ovaries should be removed; that on account of their diseased condition they were useless so far as their functions as generative organs were concerned, and hence the woman was better without them. Many of these operators have not taken into consideration the question of whether the function of menstruation should or should not be retained by the preservation of healthy ovaries or parts of healthy ovaries.

Now a third class is coming forward who say: First, do all that can be done by medicinal and local treatment; then when operation is necessary, do all you can to preserve the function of menstruation and the possibility of conception.

Dr. Gardner then quoted the opinions of several eminent authorities as to the effect of castration upon woman, and made a review of a number of cases treated by these men in the conservative manner, concluding with the following statements:

1. Women are better mentally and physically for the maintenance of menstruation and ovulation up to the period of nature's menopause.

2. Resection of the Fallopian tube when it does not contain pus is a practical operation.

3. When the tube is so diseased as to demand removal, the ovary being healthy, the tube should be removed and the ovary left.

4. Cysts of the ovary do not demand its removal in all cases; good results have been reported from resection.

5. Adhesions do not demand removal of tubes and ovaries, unless they are so dense that in breaking them the appendages are injured.

6. In all cases of chronic disease of the tubes the uterus should be curetted and packed with gauze.

7. In enucleation of fibroid tumors leaving the ovaries is better than to remove the ovaries and leave the fibroid.

8. Conservative operations upon the appendages instead of rendering the patients hopelessly barren, have cured a considerable number of cases of sterility.

DR. W. P. CHUNN: I believe in conservative methods, and would try mild means before proceeding to radical operations. When the case is such as to demand a laparotomy, it is usually necessary to remove the appendages.

DR. B. B. BROWNE: Conservatism seems to be the order of the day. I am satisfied that curetting and drainage frequently bring about such good results as to remove the necessity for laparotomy. It is always best, if conditions permit, to try the easier method first.

DR. J. H. BRANHAM: It is best to try easy methods and to save all we can to the woman, but in cases requiring laparotomy, conservatism of operation may be carried too far. I can hardly conceive of the epithelium being restored in a tube which has been the seat of suppurative inflammation. The chances of restoring the tube to its natural functions by delay are very slim. If you are sure you have pus tubes, it is best to operate. Delay only leaves you in danger of extension.

The typical ovarian cyst, with chocolate colored fluid and hypertrophied tissue, is essentially cancerous and is only quiet for a time. If you leave a piece of this you are pretty sure to have sooner or later general infection. As for ovary with multiple cysts the pain is very great and if you must operate for relief it is best to take out the whole ovary, otherwise you only leave chance for further cyst formation.

As for the operation unsexing a woman, it is not a universal opinion that there is such a great change.

DR. GARDNER, in closing the discussion, took exception to Dr. Branham's remarks about the cancerous character of ovarian cysts.

DR. HIRAM WOODS, in his paper "Is Ophthalmia Neonatorum Always Curable?" said: Last December at the Presbyterian Eye and Ear Hospital a baby came out of an attack of ophthalmia neonatorum with scarred cornea. Both eyes will have useful but never perfect sight. In the summer of '92 I saw another case with one cornea badly damaged. In both cases the home cleaning was bad and in one the mother failed for several successive days to bring the child to hospital for silver treatment. In the Transactions of the American Ophthalmological Society for '93 is an account of a case lost by Dr. Randall, of Philadelphia, despite the most careful treatment and it reminds me of a baby I saw three or four years ago, blind, who had been treated correctly from the beginning. In the light of these cases I have reviewed my experience and observation of this disease with special reference to the question, is it always curable? The experience of Dr. Randall and others compels a negative answer. But such cases are exceptional. The vast majority can be cured by routine treatment in from three to fourteen days. A few cases are not helped by cleaning and the one or two per cent. silver solution. Why? Frequently I believe because of rough handling. The disease is seen usually among the poor, trained nurses cannot be employed and half-hourly cleansing is necessary. Unskillful attempts to clean the conjunctival sac often result in injury. Sometime cleanliness and daily use of one or two per cent. of sol. silver seem to have no effect upon a case. Should the silver be discontinued or employed in stronger form? As nearly as I can give the clinical condition demanding the discontinuance of silver it is: conjunctival purulency, unaccompanied by lid infiltration, papillary swelling of conjunctiva and the deep red color seen in severe cases. There comes a time in a certain percentage of cases when, although purulency continues, nitrate of silver will not cure. In these cases it makes little difference what you use besides cleanliness, so the silver is stopped.

As for stronger silver applications, they are only *rarely* called for, and should never be used save by a skilled hand. The symptoms calling for it are: profuse discharge of pus, swollen infiltrated lids, the rugous hypertrophied conjunctiva and the failure to respond to a weak solution. Children of premature birth, those relying on artificial food or those of weak constitution form a class of cases in which prognosis is grave. Finally I wish to protest against the use of cocaine in this disease to allay pain. Such practice is a deliberate courting of trouble.

DR. E. J. BORNSTEIN: I have met with one case which resulted badly despite the fact that I pursued the usual treatment and had trained nurse to look after detail.

DR. R. L. RANDOLPH: I have always noticed that the disease is most virulent in ill fed or diseased children. I have always looked upon the affection as a modified form of the gonorrheal ophthalma of adults, and agree with Dr. Woods as to the treatment.

DR. R. B. NORMENT: It strikes me that the cardinal point is to get at the treatment early. I have kept the eyes clean and used sol. bichloride 1 to 6000 freely. I do not consider silver nitrate safe except in skilled hands. Bichloride can be used by any one without danger and my results have been good.

DR. H. PRIEDENWALD: In some patients the disease is very slight and will get well without treatment other than cleanliness. It is surprising to me that the cornea is not more frequently attacked, when we consider its likelihood in the similar disease of adults. Silver nitrate should be applied to the lids. *Dropping* it upon the eye may cause opacity by cauterization. Bichloride has been tried often but does not compare in value with silver. In test cases where it was used in one eye and silver in the other, the latter produced the most prompt and best results by far.

DR. WOODS: Eyes have been lost by some of the most skillful oculists, as Knapp, Andrews, Randall, etc. The vast majority of cases are curable by early and proper treatment.

In Dr. Norment's cases the [point was cleanliness. I think 1 to 6000 bichloride is no better than water. I doubt if that solution would kill the gonococcus were it present. The reason for not using cocaine is that it produces exfoliation of the cornea, and thus opens the way for the germs. It is pretty generally recognized now that other secretions than gonorrheal may cause ophthalmia neonatorum.

H. O. REIK, M.D., Secretary.

*525 N. Howard St.*

